# POLYGENETIC SCORE IS ASSOCIATED WITH T2D; EVIDENCE FROM THE HEALTH AND RETIREMENT STUDY

**DOI:** 10.1093/geroni/igz038.3264

**Published:** 2019-11-08

**Authors:** Fadi Youkhana, Yanyan Wu, Mika Thompson, Catherine M Pirkle

**Affiliations:** University of Hawai’i at Mānoa, Honolulu, Hawai’i, United States

## Abstract

Type 2 diabetes (T2D) is a complex chronic disorder influenced by genetic and environmental factors. Studies that use a combined polygenetic score (PGS), calculated based on the number of risk alleles an individual may have, are rarely applied to a representative national sample. We used data from the Health and Retirement Study (HRS), a nationally representative study of older U.S. adults 50-years or older to examine the impact of PGS and behavioral risk factors (education, poverty ratio, BMI, smoking status, alcohol consumption and physical activity) with incident T2D. We used ethnic-straitifed Poisson generalized estimating equation (GEE) models with robust standard errors to estimate prevalence ratios (PRs) and risk ratios (RRs). Our sample included genotyped Black (N=2,823) and White (N=11,178) men and women.The highest PRs for T2D were among those in the 5th PGS quintile in both Whites (PR=2.24, 95%CI 1.89, 2.65, P-value <0.0001) and Blacks (PR=1.73, 95%CI 1.28,2.33, P-value 0.0003). The highest risk for T2D was among obese Whites (RR=3.35, 95%CI 2.93,3.82, P-value <0.0001) and Blacks (RR=1.60, 95%CI 1.28, 2.00, P-value <0.0001). Our findings found associations between PGS and T2D as well as some lifestyle factors among both Black and White individuals in a nationally representative sample with similar patterns in age, physical activity and poverty ratio. Our study supports the importance of including modifiable and non-modifiable life-style factors in the analysis of risk alleles for T2D to continue addressing the disparities between T2D risk between race/ethnicity groups

